# Expression of *Stipa purpurea* *SpCIPK26* in *Arabidopsis thaliana* Enhances Salt and Drought Tolerance and Regulates Abscisic Acid Signaling

**DOI:** 10.3390/ijms17060966

**Published:** 2016-06-22

**Authors:** Yanli Zhou, Xudong Sun, Yunqiang Yang, Xiong Li, Ying Cheng, Yongping Yang

**Affiliations:** 1 Key Laboratory for Plant Diversity and Biogeography of East Asia, Kunming Institute of Botany, Chinese Academy of Sciences, Kunming 650201, China; zhouyanli@mail.kib.ac.cn (Y.Z.); sunxudong@mail.kib.ac.cn (X.S.); yangyunqiang@mail.kib.ac.cn (Y.Y.); lixiong@mail.kib.ac.cn (X.L.); 2 Plant Germplasm and Genomics Center, the Germplasm Bank of Wild Species, Kunming Institute of Botany, Chinese Academy of Sciences, Kunming 650201, China; 3 University of Chinese Academy of Sciences, Beijing 100049, China; 4 Institute of Tibetan Plateau Research at Kunming, Kunming Institute of Botany, Chinese Academy of Sciences, Kunming 650201, China; 5 College of Horticulture and Landscape, Yunnan Agricultural University, Kunming 650201, China; shidagonglao@gmail.com

**Keywords:** CIPK, *Stipa purpurea*, salt, drought, ABA, ROS

## Abstract

*Stipa purpurea* (*S. purpurea*) is the dominant plant species in the alpine steppe of the Qinghai-Tibet Plateau, China. It is highly resistant to cold and drought conditions. However, the underlying mechanisms regulating the stress tolerance are unknown. In this study, a *CIPK* gene from *S. purpurea* (*SpCIPK26*) was isolated. The *SpCIPK26* coding region consisted of 1392 bp that encoded 464 amino acids. The protein has a highly conserved catalytic structure and regulatory domain. The expression of *SpCIPK26* was induced by drought and salt stress. *SpCIPK26* overexpression in *Arabidopsis* *thaliana* (*A. thaliana*) plants provided increased tolerance to drought and salt stress in an abscisic acid (ABA)-dependent manner. Compared with wild-type *A. thaliana* plants, *SpCIPK26*-overexpressing plants had higher survival rates, water potentials, and photosynthetic efficiency (*F*v/*F*m), as well as lower levels of reactive oxygen species (ROS) following exposure to drought and salt stress. Gene expression analyses indicated stress-inducible genes (*RD29A,* *RD29B*, and *ABF2*) and a ROS-scavenger gene (*CAT1*) were upregulated in *SpCIPK26*-overexpressing plants after stress treatments. All of these marker genes are associated with ABA-responsive *cis*-acting elements. Additionally, the similarities in the gene expression patterns following ABA, mannitol, and NaCl treatments suggest *SpCIPK26* has an important role during plant responses to drought and salt stress and in regulating ABA signaling.

## 1. Introduction

Drought and salinity are two major abiotic stresses that inhibit plant growth and development [1,2,3]. Global estimates indicate more than 1/3 of irrigated lands are affected by drought and salt stress, which threaten sustainable crop production [4]. Exposure to drought and saline conditions can lead to water deficit and oxidative stress [2]. However, plants are protected by the expression of various stress-related genes to synthesize hormones (e.g., abscisic acid (ABA)) and regulatory proteins to cope with drought and salt stress [5]. The ability of plants to respond to stress and induce the production of downstream target proteins is particularly important for immobile plants [6].

Calcium (Ca^2+^) is a ubiquitous second messenger with important functions in diverse biological processes. The steady-state cytosolic free Ca^2+^ concentration is lower than 200 nM. However, it can transiently increase following exposure to certain stimuli [7], thus generating the so-called “Ca^2+^ signature”, which exhibits distinct spatial and temporal expression patterns. To initiate specific stress responses, the Ca^2+^ signal is recognized by Ca^2+^ sensors in highly ordered cascades. The Ca^2+^ sensors mainly consist of calmodulins (CaMs), calmodulin-like proteins, calcineurin B-like proteins (CBLs), and calcium-dependent protein kinases [8]. The CaMs and CBLs merely act as sensor relays because they do not have an intrinsic catalytic function [9]. Unlike CaMs, which interact with various target proteins to relay Ca^2+^ signals [10], CBLs interact with a specific group of sucrose non-fermenting-related kinases called CBL-interacting protein kinases (CIPKs) to transmit signals to downstream targets [11]. Generally, CIPKs contain a catalytic kinase loop and a regulatory FISL/NAF motif at their N and C termini, respectively [12]. Recently, a newly identified protein–phosphate interaction domain in a CIPK was revealed to interact with the 2C-type protein phosphatase, leading to crosstalk between the Ca^2+^ signaling pathway and signaling molecules such as abscisic acid (ABA) [13]. In the absence of stress, the FISL/NAF motif plays an auto-inhibitory role by inactivating the kinase domain of CIPKs. However, the perception of certain stress signals can induce CBLs to bind to the FISL/NAF motif and prevent it from inhibiting CIPK activity [14].

To date, 26 *Arabidopsis thaliana* CIPKs, 33 *Oryza sativa* CIPKs, and 27 *Populus trichocarpa* CIPKs have been identified in genomic investigations [15,16], suggesting complicated networks are involved in the interactions between CIPKs and their respective CBL partners. For example, in *A. thaliana*, SOS3 (CBL4) interacts with SOS2 (CIPK24) to mediate Na^+^ efflux by phosphorylating the Na^+^/H^+^ exchanger SOS1 [17,18]. Additionally, the overexpression of an apple SOS2-like protein increased salt tolerance in apple and tomato plants [19]. AtCIPK6 functions in plant responses to osmotic stress and ABA [20]. AtCIPK1 is involved in ABA-dependent and -independent abiotic stress signaling processes by interacting with different CBLs [21]. Despite considerable efforts in characterizing CIPKs, most of the relevant studies focused on model plant species lacking the genetic basis to survive in harsh environments.

*Stipa purpurea* (*S. purpurea*) is an endemic plant species that is widely distributed in the alpine steppe of the Qinghai-Tibet Plateau [22]. It is traditionally used as an important forage crop because of its enriched nutrient contents and ability to survive in adverse conditions (*i.e.*, low temperature, strong UV-B radiation, and drought) [23,24]. The signaling pathway of this species has not been fully characterized, however CIPK was postulated to play roles in its acclimation. In this study, a CIPK gene (*i.e.*, *SpCIPK26*), differently expressed along a drought gradient in previous transcriptome analysis [25], was isolated from *S. purpurea* and analyzed by ectopic expression in *A. thaliana* plants. Our results suggest *SpCIPK26* is responsive to salt and drought stress, and regulates ROS homeostasis and gene expression by crosstalk with ABA signaling.

## 2. Results

### 2.1. Isolation and Characterization of the Stipa purpurea SpCIPK26 Gene

**Figure 1:**
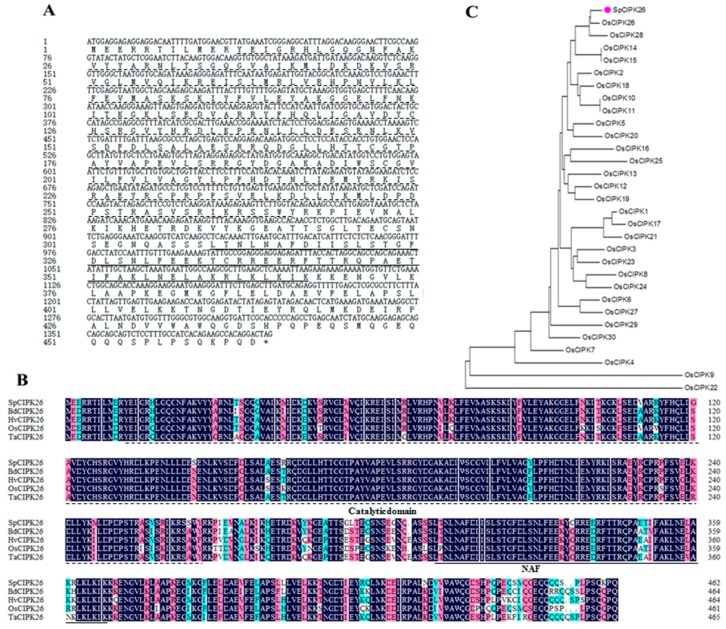
Sequence analysis of *SpCIPK26*. (**A**) Nucleotide and deduced protein sequence of *SpCIPK26*. Amino acids underlined with solid lines represent NAF domain and marked with dashed line represents catalytic domain; (**B**) alignment of the deduced protein sequence of SpCIPK26 and its homologous amino acids from other plant species. Alignments were performed in MEGA6. Identical amino acid residues are shown in indigo background, amino acid with larger than 75% and 50% identity are shown in cyan and pink, respectively; (**C**) phylogeny of SpCIPK26 and CIPK members of *Oryza sativa*. Pink dot means SpCIPK26. The phylogenetic tree was constructed in MEGA6 using the putative amino acid sequences.

The *SpCIPK26* gene contains a 1392-bp open reading frame. The encoded protein consists of 464 amino acids (Figure 1A), with a calculated molecular mass of 52.91 kDa and a predicted pI (isoelectric point) of 8.97. Multiple sequence alignments revealed that SpCIPK26 contains a conserved catalytic domain at the N-terminus, and a canonical NAF domain at the C-terminus. These features are similar to those of CIPK26 from *Brachypodium distachyon* (BdCIPK26; GI: 721653073), CIPK26 from *Triticum aestivum* (TaCIPK26; GI: 764399140), NIASHv2081P15 from *Hordeum vulgare* (HvCIPK26; GI: 326519455), and CIPK26 from *Oryza sativa* ssp. japonica (OsCIPK26; GI: 189099628) (Figure 1B). Phylogenetic analyses indicated that SpCIPK26 clusters most closely with OsCIPK26 and OsCIPK28 (Figure 1C).

**Figure 2:**
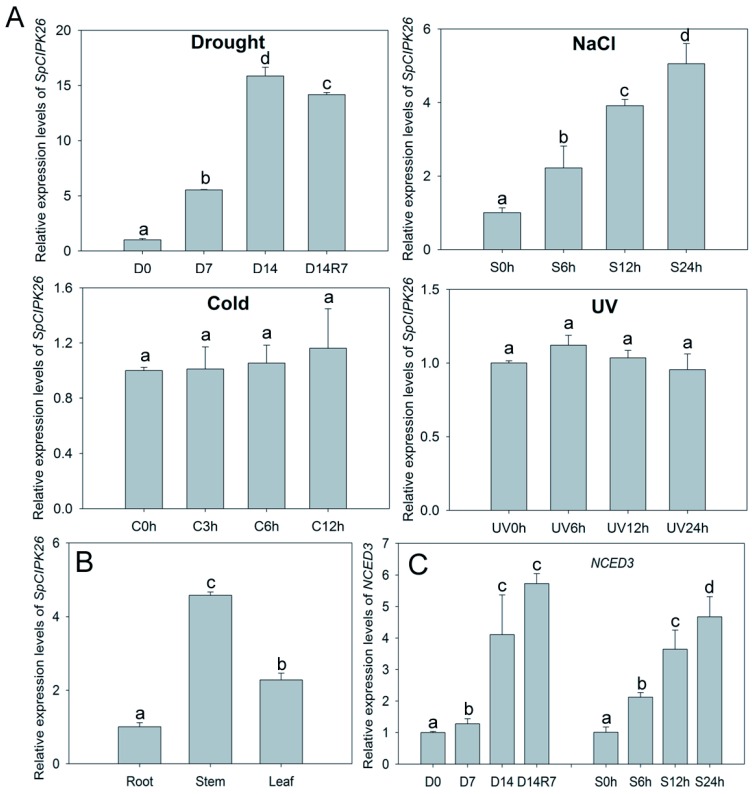
Expression pattern of *SpCIPK26* in *S. purpurea* (*Stipa purpurea*). (**A**) Relative expression levels of *SpCIPK26* in *S. purpurea* under abiotic stress. Drought stress was conducted by withholding water (D0) for seven days (D7), 14 days (D14) and then rehydrated for another seven days (D14R7). Salt treatment was conducted by irrigating three-week-old *S. purpurea* seedlings with 150 mM NaCl and samples were taken at indicated time. S suffixed with time point (*i.e*., S0h) means *S. purpurea* seedlings treated by salt for specific time. For cold stress, three-week-old *S. purpurea* seedlings were placed at 4 °C. UV treatment was imposed by irradiating ultraviolet lamp at the intensity of 8 μW/cm^2^; (**B**) transcriptional level of *SpCIPK26* in different tissues. The expression level in roots was normalized as control; (**C**) expression of *SpNECD3* in response to drought and salt treatment. All treatments were replicated three times biologically. Error bar denotes standard deviation, and different letters above the bars indicate significant difference of pair-wise comparison between different treatment times at *p* < 0.01.

As *S. purpurea* is well accustomed to the extreme drought, cold, and high UV conditions in the Qinghai-Tibet Plateau, real-time quantitative reverse transcription polymerase chain reaction (qRT-PCR) was used to investigate expression profile of *SpCIPK26* to these environmental signals at transcription level. The results showed that *SpCIPK26* was significantly upregulated by drought and salt treatment, while showed no significant response to cold and UV treatment (Figure 2A), implying that *SpCIPK26* probable play a role in drought and salt tolerance. For the tissue-specific analysis, *SpCIPK26* was found to mainly express in stem and leaf, compared with root expression level (Figure 2B). Additionally, *SpNCED3* responsible for ABA biosynthesis was transcriptionally upregulated in response to drought and salt treatment (Figure 2C), indicating its potential involvement in stress tolerance.

### 2.2. Overexpression of SpCIPK26 Enhances Tolerance to Drought and Salt Stress in Arabidopsis thaliana

**Figure 3:**
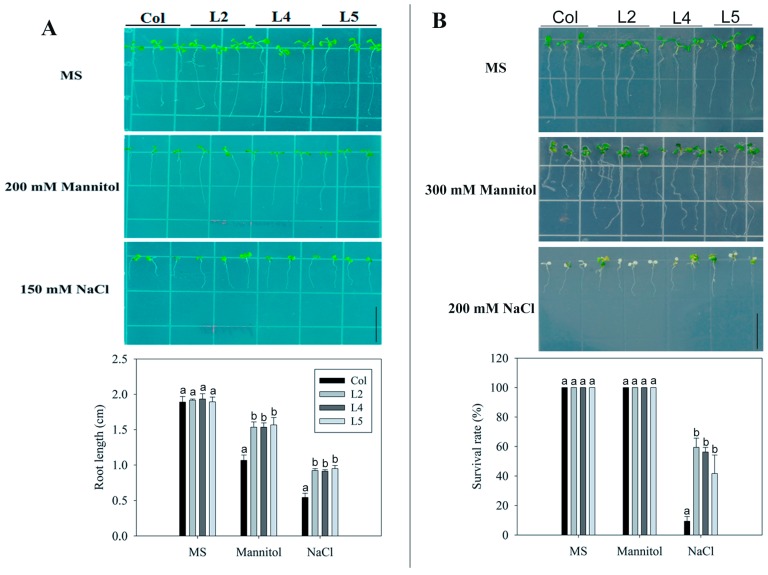
Phenotypic analysis of *SpCIPK26*-overexpressing (OXP) and WT in response to drought (mannitol) and salt (NaCl) stress. (**A**) Primary root length of transgenic plants under drought and salt stress. Seeds were grown on 1/2 Murashige and Skoog (MS) medium for 10 days and that containing 200 mM mannitol or 150 mM NaCl; (**B**) Survival rate of transgenic plants under drought and salt stress. Seedlings germinated on 1/2 MS medium for five days, then transferred to mediums containing 200 mM NaCl for 3 days or 300 mM mannitol for six days. All treatments were replicated three times biologically. Error bar denotes standard deviation, and different letters above the bars signify significant difference between wild type (control, Col) and three transgenic lines (L2, L4 and L5) at *p* < 0.01. Bars = 1 cm.

To investigate the potential involvement of *SpCIPK26* in drought and salt tolerance, the gene was inserted into the *Arabidopsis thaliana* genome. Five independent transgenic lines were identified by reverse transcription polymerase chain reaction (RT-PCR) (Figure S1). Three homozygous lines (L2, L4, and L5) were selected for further analyses. There were no significant differences of primary root length between the wild-type (WT) controls and the transgenic plants sown on 1/2 Murashige and Skoog (MS) culture medium. However, the transgenic plants were more drought and salt tolerant than the control plants sown on MS medium supplemented with 200 mM mannitol or 150 mM NaCl, respectively, with transgenic plants had significantly longer primary roots than the WT controls (Figure 3A). In another batch of post-germination test, uniform seedlings grown on 1/2 MS medium were transferred to elevated concentration of mannitol or NaCl for mortality statistics. Three days after transferring, about 45% of transgenic plants died from salt application, respectively, compared with 91% for WT (Figure 3B). However, no visible death was observed on 300 mM mannitol-containing medium, even up to the six day after transferring.

**Figure 4:**
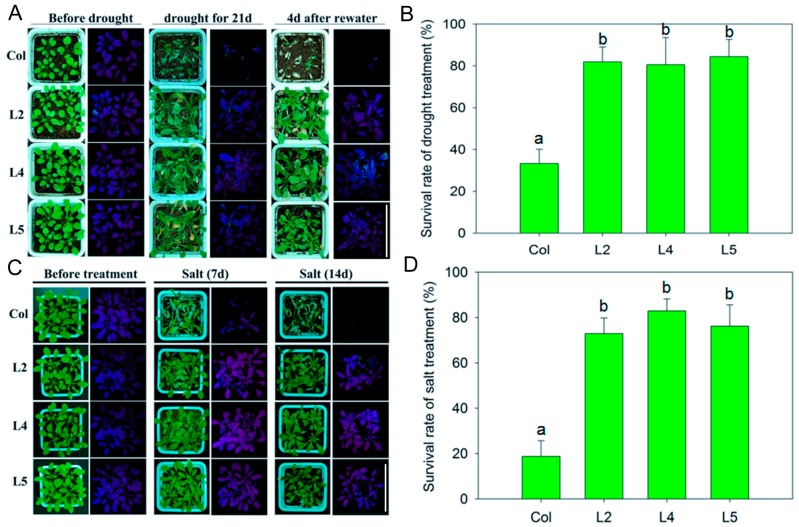
Effect of drought and salt stress on the seedling growth and survival rate of *SpCIPK26*-OXP and WT planted in soil: (**A**,**B**) phenotype and photosynthetic efficiency (*F*v/*F*m) comparison between *SpCIPK26*-OXP and WT in response to drought and salt treatment, respectively; and (**C**,**D**) survival rate of *SpCIPK26*-OXP and WT after drought and salt stress. Drought treatment was imposed by continuously withholding water to soil-grown plants and salt was done by irrigating 30 mL NaCl solution every day at the concentration of 150 mM. All treatments were replicated six times biologically. Error bar denotes standard deviation, and different letters above the bars signify significant difference between wild type (control, Col) and transgenic lines (L2, L4 and L5) at *p* < 0.01. Bar = 10 cm.

To confirm the increased drought and salt tolerance of *SpCIPK26*-overexpressing (OXP) plants in soil, one-month-old plants were deprived of water for 21 days, and irrigated with 150 mM NaCl (30 mL/pot daily for 14 days). There were no significant differences between untreated SpCIPK26-OXP and WT plants. In contrast, *SpCIPK26*-OXP plants were more drought-tolerant than WT plants. Transgenic plants remained healthy and green, while WT plants wilted following the drought treatment, as evidenced by the photosynthetic efficiency (*F*v/*F*m) values, which correspond to maximum quantum yield (Figure 4A). After rewatering for four days, only about 30% of WT plants recovered from the drought stress, while >80% of *SpCIPK26*-OXP plants survived. Additionally, the surviving *SpCIPK26*-OXP plants recovered almost completely, while only a few young leaves surrounding inflorescences survived in WT plants (Figure 4B). The *SpCIPK26*-OXP and WT plant responses following exposure to salt stress were similar to those observed after drought treatment (Figure 4C,D).

### 2.3. Overexpression of SpCIPK26 Increases Water Potential and Abscisic Acid Content and Decreases Reactive Oxygen Species Accumulation

**Figure 5:**
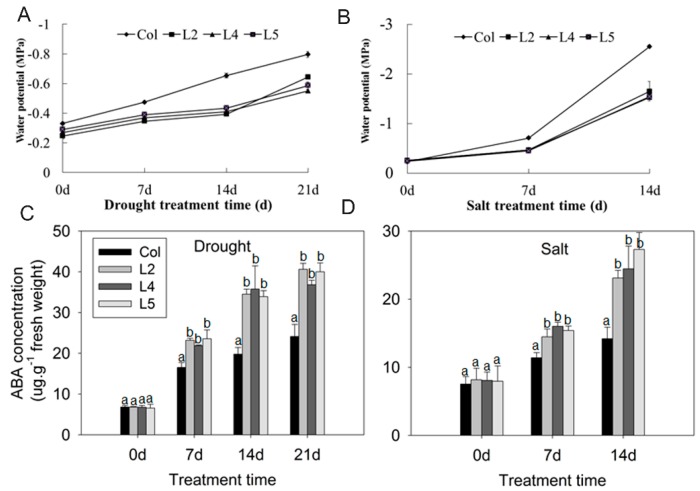
Water potential and ABA content of *SpCIPK26*-OXP and WT suffered from drought and salt treatment: (**A**,**B**) water potential of transgenic and WT plants at different drought and salt treatment time; and (**C**,**D**) stand results of ABA concentration of plant leaves stressed by drought and salt for different time. Drought was carried out by withholding water for indicated time, and salt was handled by irrigating 150 mM NaCl every day and samples were collected weekly. All treatments were replicated three times biologically. Error bar denotes standard deviation, and different letters above the bars signify significant difference between wild type (control, Col) and transgenic lines (L2, L4 and L5) at *p* < 0.01.

The fact that the *SpCIPK26*-OXP plants were more drought and salt tolerant than the WT plants prompted us to study the physiological differences between the transgenic and wild-type lines. Water potential provides information regarding the water status of plants, and is important for evaluating osmotic stress [26]. The leaf water potential was higher in *SpCIPK26*-OXP plants than in WT controls under the same drought or salt stress conditions (Figure 5A,B), implying the overexpression of *SpCIPK26* enhanced the water retention ability of the transgenic plants. Abscisic acid is a well-characterized phytohormone involved in responses to drought and salt stress [27,28]. The ABA levels were significantly higher in the *SpCIPK26*-OXP plants than in the WT controls following drought or salt treatment (Figure 5C,D), which is consistent with the drought and salt tolerant phenotype of *SpCIPK26*-OXP plants.

**Figure 6:**
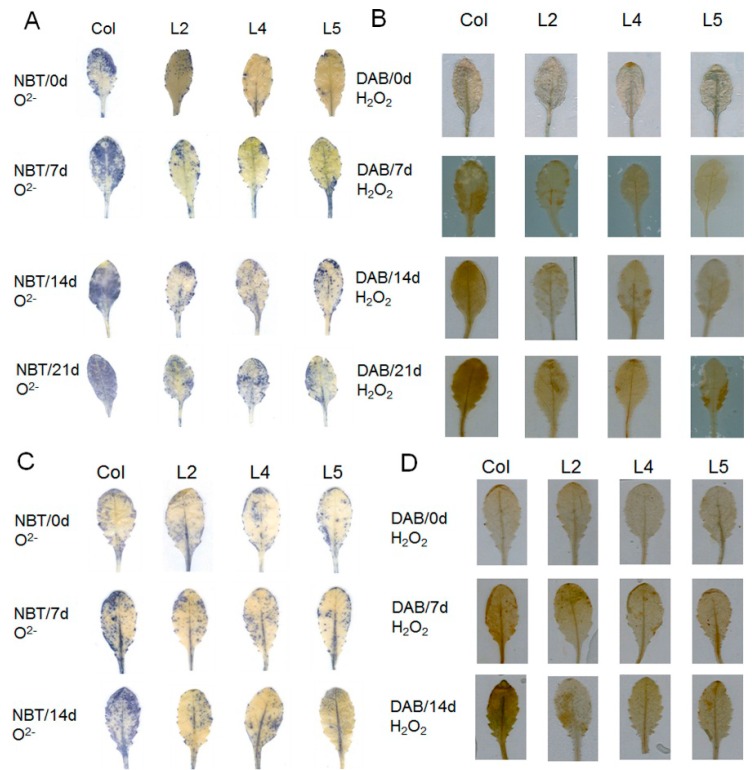
*In situ* ROS generation in *SpCIPK26*-OXP and WT subjected to drought and salt stress: (**A**,**C**) O_2_^−^ contents in plants treated with drought and salt, respectively; and (**B**,**D**) H_2_O_2_ detection of plants treated with drought and salt, respectively. Stress was processed as depicted in Figure 3. O_2_^−^ was detected by nitro-blue tetrazolium (NBT) reduction and H_2_O_2_ was detected by diaminobenzidine (DAB) at specific time points. Samples were taken on the 7th, 14th and 21st days for drought and at the 7th and 14th for salt, and samples at the start of treatments were taken as control (Col). The 4th–6th leaves were taken from every plant. All treatments were replicated three times biologically. L2, L4 and L5 mean three transgenic lines (*SpCIPK26*-OXP).

Additionally, excess reactive oxygen species (ROS) damages cell membranes through oxidations of unsaturated fatty acids [29]. The steady-state levels of two key ROS (*i.e.*, H_2_O_2_ and O^2−^) gradually increased throughout the drought and salt treatment periods in WT and *SpCIPK26*-OXP plants, but the levels were lower in the transgenic plants at all time points (Figure 6A–D). These results suggested *SpCIPK26* had a positive regulatory role during drought and salt stress responses. Conclusively, all above physiological results supported that *SpCIPK26*-OXP plants were more tolerant to drought and salt stress than the WT controls.

### 2.4. The Abscisic Acid-Dependent Signaling Pathway Contributes to the Drought and Salt Tolerance of SpCIPK26-Overexpressing Plants

**Figure 7:**
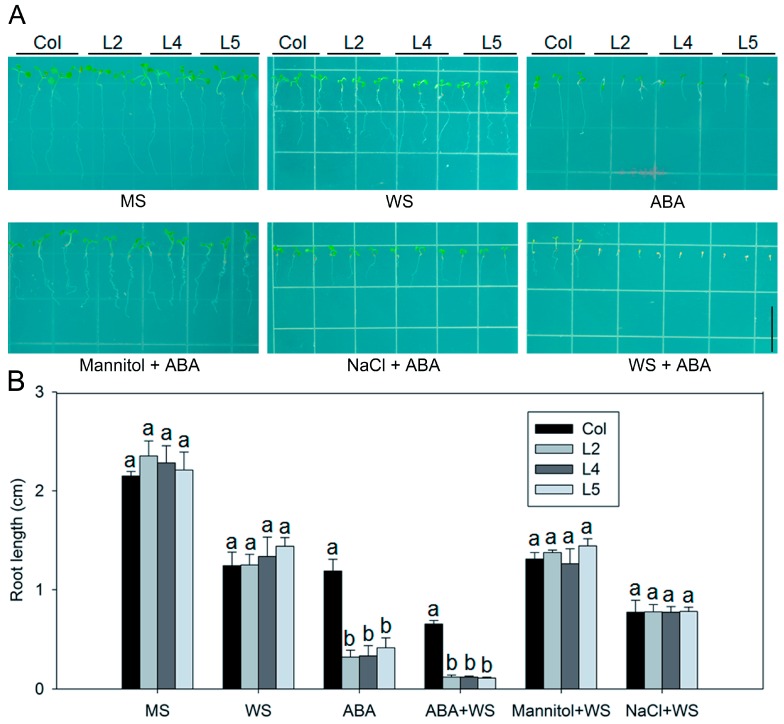

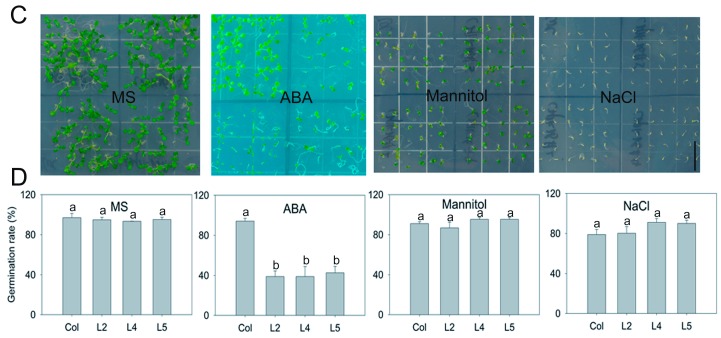
Effect of ABA on the root length, stress tolerance and germination of *SpCIPK26*-OXP. (**A**) Effect of ABA and its inhibitor sodium tungstate (WS) on the seedling growth under normal or stress condition. Photograph was taken of seven-day-old seedlings from each line; (**B**) root length of seedlings presented in (**A**); (**C**) germination of *SpCIPK26*-OXP and WT seeds sown on half MS containing 0.2 μM ABA, 200 mM mannitol and 150 mM NaCl; (**D**) Germination percentage of seedlings presented in (**C**) and the number of seedlings was counted as emergence of cotyledon. All experiments were replicated three times and each column represent means ± SD. Error bar denotes standard deviation, and different letters above the bars signify significant difference between wild type (control, Col) and transgenic lines (L2, L4 and L5) at *p* < 0.01. Bars = 1 cm.

We investigated the relationship between *SpCIPK26* and ABA signaling. There were no significant differences between *SpCIPK26*-OXP and WT plants when cultured on 1/2 MS medium with or without the addition of 0.2 mM sodium tungstate (WS, ABA biosynthesis inhibitor), although WS slightly affected growth as a chemical regent (Figure 7A). However, the roots of *SpCIPK26*-OXP plants were hypersensitive to the application of exogenous ABA, and were about 1/3 shorter than the WT roots in the presence of 0.2 μM ABA. These root effects were unaffected by sodium tungstate (Figure 7B). We also confirmed the relationship between *SpCIPK26* and ABA by observing seed germination on ABA-supplemented medium. *SpCIPK26*-OXP seeds were sensitive to exogenous 0.2 μM ABA, but not to 200 mM mannitol or 150 mM NaCl (Figure 7C). The germination rate was approximately 40% for *SpCIPK26*-OXP seeds, which was considerably lower than the 94% for WT plants under 0.2 μM ABA treatment (Figure 7D). Additionally, treatment with 0.2 mM sodium tungstate rendered the *SpCIPK26*-OXP plants almost as susceptible to drought (200 mM mannitol) and salt (150 mM NaCl) stress as the WT plants (Figure 7A). This suggested the tolerance of *SpCIPK26*-OXP plants to drought and salt stress was mediated, at least partly, by an ABA-dependent signaling pathway.

### 2.5. Upregulation of Stress-Related Genes of SpCIPK26-OXP Plants in Response to Abscisic Acid, Drought, and Salt Treatments

**Figure 8:**
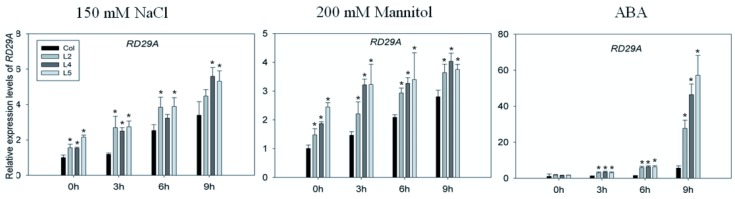

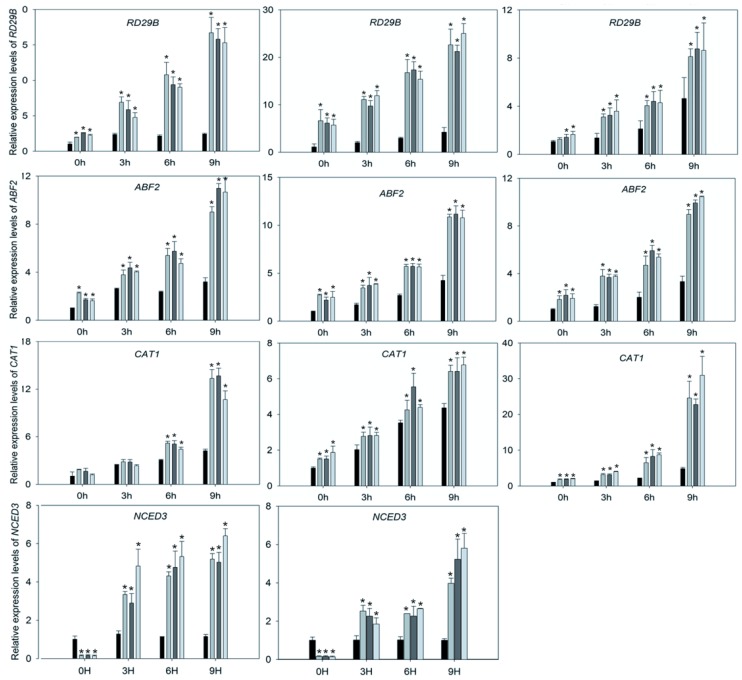
Results of qRT-PCR analysis on stress-related genes in response to ABA, mannitol and NaCl treatment. Expression profile in the first column are results of *SpCIPK26*-OXP and WT irrigated with 0.2 μM ABA, which in middle and right column are treated with 200 mM mannitol and 150 mM NaCl for 0, 3, 6 and 9 h, respectively. The *SpCIPK26*-OXP and WT cultured on 1/2 MS for two weeks were subjected to stress treatments and values of each gene detected in wild type at 0 h was standardized to 1. Amplification of UBQ10 was used at internal control. Mean values and standard deviation (error bar) were calculated from three independent experiments and asterisk (*) above the bar stand for significant difference between wild type (Col) and transgenic lines (L2, L4 and L5) at *p* < 0.01.

Because *SpCIPK26*-OXP plants were extremely sensitive to ABA during early seedling development, we analyzed the expression levels of various ABA-related stress marker genes. We focused on well-established ABA-responsive genes (*i.e*., *RD29B* and *ABF2*), a dehydration-inducible gene (*i.e.*, *RD29A*), ABA biosynthetic genes (*NCED3*) and an ROS-related gene (*i.e*., *CAT1*) in two-week-old *A. thaliana* plants treated with or without 0.2 μM ABA, and 200 mM mannitol or 150 mM NaCl. The transcript levels of all analyzed stress marker genes were higher in *SpCIPK26*-OXP plants than in WT controls, even in the absence of stress (Figure 8). Treatments with 0.2 μM ABA, 200 mM mannitol, or 150 mM NaCl resulted in gradual increases in the expression levels of these marker genes in WT and *SpCIPK26*-OXP plants. However, the expression levels in *SpCIPK26*-OXP plants were higher than in WT controls at all time points for all genes (Figure 8). These results confirmed the drought and salt tolerance of *SpCIPK26*-OXP plants at the transcript level.

## 3. Discussion

The involvement of cellular Ca^2+^ in stress signal transductions in plants has been investigated in several studies [30,31,32]. The “Ca^2+^ signature” is detected by CIPKs through interactions with CBL(s) in the form of a CBL/CIPK complex [14,33,34]. Although some *A. thaliana* AtCIPKs have been functionally characterized to impart stress tolerance, more CIPKs from other non-model plants, especially those growing in harsh habitats, need to be identified and characterized [35]. In this study, a CIPK isolated from *S. purpurea* (*i.e.*, SpCIPK26), was observed to improve drought and salt tolerance in *A. thaliana* plants.

AtCIPK26 was reported to interact with ABA-related components ABI1, ABI2 and ABI5 [36], raising the possibility that AtCIPK26 effect on the ABA mediated process. SpCIPK26 was responsive to drought gradient in our transcriptome analysis (data not show), potentially contributing to drought adaptation of *S. purpurea*. As revealed by qRT-PCR, expression of SpCIPK26 was upregulated significantly by drought and salt stress (Figure 2A). Further ectopic expression of SpCIPK26 in *A. thaliana* conferred longer primary roots and higher survival rates under drought and salt stress (Figure 3). However, SpCIPK26 mainly expressed in *S. purpurea* aboveground parts (Figure 2B), indicating its intricate regulation at whole-plant level. Abscisic acid is a versatile stress response signaling molecule that regulates pleiotropic plant responses to environmental stress via transcriptome alterations, translational regulation, or post-translational modifications, it can be synthesized *in situ* or transported to functional sites upon stress initiation [37,38]. Increased endogenous ABA levels were observed in plants treated with drought and saline conditions (Figure 5C,D). However, the ABA levels were always higher in *SpCIPK26*-OXP plants than in WT controls for all stress treatments, which is consistent with the hypersensitive phenotype observed during the germination of *SpCIPK26*-OXP seeds (Figure 7C). The addition of sodium tungstate to inhibit the biosynthesis of endogenous ABA eliminated the superior growth of *SpCIPK26*-OXP plants over the WT controls on mannitol- or salt-supplemented MS media. This indicated ABA positively contributed to drought and salt tolerance in *SpCIPK26*-OXP plants.

Abscisic acid signaling activates downstream substrates in diverse ways [39,40]. The ABA-responsive *cis*-acting element (ABRE)-binding transcription factor (ABF) is a conserved *cis*-element in the promoters of ABA-inducible genes, with important functions in stress response pathways [41]. In this study, two dehydration-inducible genes (*i.e.*, *RD29A* and *RD29B*) were upregulated more in *SpCIPK26*-OXP plants following ABA, mannitol, and NaCl treatments than in WT controls (Figure 8). The promoter region of *RD29A* contains at least two *cis*-acting elements, namely DRE (dehydration responsive element) and ABRE, while the *RD29B* promoter contains only ABRE [42]. The increase in the expression of *RD29B* is higher (*i.e.*, 10–30-fold) than that of *RD29A* (*i.e.*, 2–4-fold) following drought treatment, suggesting the importance of ABA in *SpCIPK26*-OXP plants. The ABF2 transcription factor (basic leucine zipper) binds to the ABRE motif in the promoter region of ABA-inducible genes. Additionally, the ABA-dependent phosphorylation of a rice ABF (TRAB1) at Ser102 is crucial for activating the transcription factor [43]. Constitutive expression of its homologous gene (*i.e.*, *AhAREB1*) in *Arachis hypogaea* led to increased drought tolerance via the upregulation of several ABA- and drought-related genes [44]. In this study, we observed that ABF2 expression increased in *SpCIPK26*-OXP plants after ABA, mannitol, or salt treatments. When ABA was applied exogenously to plants, the stress marker gene expression levels in *SpCIPK26*-OXP plants were higher than those of WT controls. This suggests that ABA functions upstream of SpCIPK26. However, SpCIPK26 was thought to mediate ABA biosynthesis based on the higher ABA contents in *SpCIPK26*-OXP plants than in WT controls. If this were the case, the effects of ABA would increase indefinitely and uncontrollably. Because of the dual role of ABA (*i.e.*, protection from stress and growth retardation), ABA activities need to be tightly regulated. Fortunately, recent studies on the ABA signaling pathway revealed the ABA receptor proteins (*i.e.*, PYR/PRL/RCAR) bind to 2C-type protein phosphatases, which may result in a feedback mechanism for ABA to control SpCIPK26 activity via dephosphorylation [45,46]. Because ABA accumulates in response to drought and salt stress, the similar expression patterns of stress-related genes following ABA and mannitol or NaCl treatments supports the hypothesis that SpCIPK26 mediates drought and salt tolerance through a crosstalk with the ABA-dependent pathway.

The increased drought and salt stress tolerance of *SpCIPK26*-OXP plants was also reflected by several physiological factors. Water potentials have been commonly used in studies examining drought, salt, and cold-related stress [47,48,49]. Compared with WT controls, the *SpCIPK26*-OXP plants retained more water following drought and salt treatments (Figure 8). The enhanced water retention capability of *SpCIPK26*-OXP plants enabled cells to maintain normal pressure levels and avoid cellular damage. The accumulation of excess ROS under stress conditions is common among living organisms, and damages lipids, nucleic acids, proteins, and especially membrane systems [50,51]. However, ROS accumulation depends on the homeostasis between ROS-producing and -scavenging activities [52,53]. In a previous study involving a heterologous expression system, AtCIPK26 suppressed the production of ROS by AtRbohF [54]. Nevertheless, SpCIPK26 also served as a negative regulator of ROS, as indicated by the higher H_2_O_2_ and O^2−^ contents in WT controls than in *SpCIPK26*-OXP plants under the same environmental conditions. It is noteworthy that in addition to its potential regulation of RbOH activities, SpCIPK26 also upregulates *CAT1* expression, leading to the conversion of H_2_O_2_ to oxygen and water (*i.e.*, elimination of ROS) (Figure 8). However, the *CAT1* promoter in maize (monocot) and *A. thaliana* (dicot) plants contains a G-box or ABRE sequence [55,56,57,58].

Overall, our observations indicate SpCIPK26 increases the tolerance of *A. thaliana* plants to drought and salt stress, mainly through an ABA-related signaling pathway. However, the biological pathway involved has not been fully characterized. Further analyses of downstream target(s) are needed to increase our understanding of how SpCIPK26 influences tolerance to drought and salt stress.

## 4. Materials and Methods

### 4.1. Growth Conditions and Stress Treatments

*Stipa purpurea* (*S. purpurea*) seeds were collected from the same population in the eastern part of the Qinghai-Tibet Plateau. Germinated *S. purpurea* seedlings were transplanted into small pots (10 cm deep) filled with nutrient soil, with 30 seedlings per pot. The seedlings were grown in a phytotron (16-h light/8-h dark photoperiod) for 3 weeks, and then treated with one of four stresses. Drought conditions were simulated by depriving seedlings of water for 7 or 14 days, and then rewatering as normal for 7 days. Seedlings experienced salinity stress with daily treatments of 30 mL 150 mM NaCl for 14 days. Seedlings were also exposed to cold stress (4 °C) and ultraviolet (UV) radiation (8 μW/cm^2^). Plants were sampled at indicated time points for each treatment, and then frozen in liquid nitrogen.

### 4.2. SpCIPK26 Cloning and Sequence Analysis

Total RNA was extracted from *S. purpurea* seedlings using TRIzol reagent (Invitrogen, Carlsbad, CA, USA). Based on the candidate expressed sequence tags from the library of drought-inducible *S. purpurea* cDNA, the following primers were designed for 3′ and 5′ rapid amplification of cDNA ends: *SpCIPK26*-F1 (5′-CTGAGGGAAATCAAGCGTC-3′) and *SpCIPK26*-R1 (5′-GACTTTTAAGGTTTTCACTCTCG-3′). The putative full-length *SpCIPK26* cDNA sequence was amplified using the following gene-specific primers: *SpCIPK26*-F (5′-ATGGAGGAGAGGAGGACAAT-3′) and *SpCIPK26*-R (5′-TAGTCCTGTGGCTTCTGTG-3′). The purified amplification product was inserted into the pMD18-T vector (TaKaRa, Dalian, China) according to the manufacturer’s instructions.

After confirming the accuracy of the inserted cDNA sequence, the *SpCIPK26* coding sequence was used to search nucleotide databases using the BLASTN tool from the National Center for Biotechnology Information website (http://www.ncbi.nlm.nih.gov/). SpCIPK26 homologs from other gramineous species were used for multiple sequence alignments of the encoded proteins. The SpCIPK26 functional structures were predicted using the SMART prosite server (http://smart.embl-heidelberg.de/). To predict biological functions, *Oryza sativa* CIPK protein sequences were used to construct a phylogenetic tree for SpCIPK26 with MEGA6.0 software.

### 4.3. SpCIPK26 Expression Vector for Arabidopsis thaliana Transformation

The complete *SpCIPK26* coding sequence was amplified with primers to introduce *Nco*I and *BamH*I sites in the forward and reverse directions. The purified amplified product was cloned in frame into the *Nco*I and *BamH*I sites of the *pRI101GFP* vector under the control of the CaMV 35S promoter. The *pRI101-SpCIPK26GFP* construct was inserted into cells of *Agrobacterium tumefaciens* strain GV3101, which were then used to transform *A. thaliana* plants according to the floral dip method [53]. Transgenic plants carrying the *pRI101-SpCIPK26GFP* construct were screened on 1/2 MS medium containing 50 µg/mL kanamycin, and then transferred to soil. Plants were grown to maturity to harvest seeds and confirm the presence of *SpCIPK26* by RT-PCR. Homozygous transgenic seeds from the T_2_ and T_3_ generations underwent segregation analysis with selective media and PCR.

### 4.4. Germination and Phenotype Assays

Homozygous seeds from *SpCIPK26*-OXP plants were vernalized at 4 °C for 3 days. The seeds were surface sterilized in 20% (*v*/*v*) sodium hypochlorite for 10 min and rinsed five times with sterilized water. To determine germination rates, seeds were added to solid 1/2 MS medium containing 0.2 μM ABA. Cotyledon emergence and fresh weight were assessed 4 and 7 days later, respectively. Tolerance to drought and salt stress at the seedling stage was analyzed on 1/2 MS medium supplemented with 200 mM mannitol and 150 mM NaCl, while the tolerance at the post-emergence stage was determined in 1-month-old soil-grown plants. We collected samples from treated plants every week to test other parameters associated with stress tolerance, including *F*v/*F*m ratio, ABA content, and water potential. All reagents were purchased from Sangon Biotech Co., Ltd. (Shanghai, China) unless otherwise indicated.

### 4.5. Quantitative Reverse Transcription Polymerase Chain Reaction

To investigate the expression levels of specific genes, quantitative RT-PCR (qRT-PCR) analyses were conducted using total RNA isolated from *S. purpurea* or *A. thaliana* plants. The purity and quantity of the isolated RNA were determined by analyzing samples at 260, 230, and 280 nm wavelengths with an ND-1000 spectrophotometer (NanoDrop, Santa Clara, CA, USA). First-strand cDNA was synthesized from a 2 μg DNA-free total RNA template using the GoScript Reverse Transcription System (Promega, WI, USA). The PCR amplifications were completed in 20 μL samples using the TransStart Top Green qPCR SuperMix (TransGen, Beijing, China) and the ABI 7500 Real-Time PCR system (Applied Biosystems, USA). The PCR program was as follows: 94 °C for 30 s; 40 cycles of 94 °C for 5 s, 55–60 °C for 15 s, and 72 °C for 34 s; and 72 °C for 10 min. The *SpACT2* and *AtUBQ10* genes were used as internal controls for *S. purpurea* and *A. thaliana*, respectively. The relative expression levels of target genes were calculated by first normalizing against the internal control, and then standardizing with the treatment control using the 2^−ΔΔ*C*t^ method [59]. The qRT-PCR primer sequences are listed in Table S1.

### 4.6. Fv/Fm and Water Potential

*F*v/*F*m of whole leaf was photographed as described in [60]. After been subjected to dark adaption for half hour, whole plants was exposed to 0.8-s pulsed light of 4000 μmol·s^−1^·m^−2^ for maximum fluorescence (*F*m) recording and to the weak measuring pulses for the minimal fluorescence (*F*o) recording.

Water potential of plant leaves was measured by WP4C dewpoint potentiometer. Briefly, fresh leaves were sampled and sliced into pieces quickly, then transferred to the chamber cavity. When difference between sample temperature (Ts) and block chamber temperature (Tb) less than −0.5 °C, chamber was sealed to measure leaf water potential.

### 4.7. Abscisic Acid Contents

Abscisic acid contents were analyzed by high-performance liquid chromatography–tandem mass spectrometry according to a published procedure [61]. Exactly 0.1 g frozen sample powder was treated with 1 mL ethyl acetate spiked with 40 ng D6-ABA as an internal control. Samples were mixed for 10 min, and then centrifuged (14,000× *g*) for 10 min at 4 °C. The supernatants were transferred to new tubes and evaporated in a vacuum concentrator. Dried samples were redissolved in 550 μL 70% (*v*/*v*) methanol, mixed, and centrifuged (14,000× *g*) for 15 min at 4 °C to remove impurities. The upper liquid layer was transferred to a vial and analyzed using the 1200 L LC–MS system (Shimadzu, Kyoto, Japan).

### 4.8. In Situ Reactive Oxygen Species Detection

The generation of ROS in SpCIPK26-OXP and WT plants under drought and salt stress conditions was investigated using nitro-blue tetrazolium (NBT) and the diaminobenzidine (DAB) reduction method according to published procedures [60], with slight modifications. Briefly, leaf samples were infiltrated with NBT (10^–2^ M) for 2 h, and then treated with 95% ethanol to detect O_2_^−^ production. Similarly, leaf samples were treated with 1 mg/mL DAB to analyze H_2_O_2_ content.

## Supplementary Materials

Supplementary materials can be found at http://www.mdpi.com/1422-0067/17/6/966/s1.

## Abbreviations

CIPK	Calcineurin B-Like Protein-Interacting Protein Kinase
ROS	Reactive Oxygen Species
