# Supplementary material for: Expression of Stipa purpurea SpCIPK26 in Arabidopsis thaliana Enhances Salt and Drought Tolerance and Regulates Abscisic Acid Signaling
Source: Int J Mol Sci. 2016 Jun 22;17(6):966. doi: 10.3390/ijms17060966 (PMC4926498; doi:10.3390/ijms17060966)
Supplement: Supplementary file 1 [file ijms-17-00966-s001.pdf]

Supplementary Materials: Expression of *Stipa purpurea SpCIPK26* in *Arabidopsis thaliana* Enhances Salt and Drought Tolerance and Regulates Abscisic Acid Signaling

Yanli Zhou, Xudong Sun, Yunqiang Yang, Xiong Li, Ying Cheng and Yongping Yang

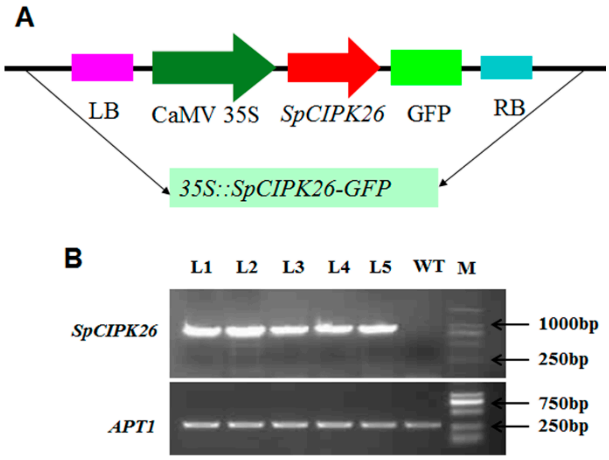

Figure S1. Generation and authentication of *SpCIPK26* transgenic *Arabidopsis thaliana* seedlings. (**A**) Construct diagram of overexpressed *SpCIPK26*. The expression of *SpCIPK26* cDNA insert is under the control of cauliflower mosaic virus 35S promoter, and introduced by *Sal*I and *Eco*RI endonuclease digestion. LB, left border; RB, right border; GFP, green fluorescence protein; (**B**) the authentication of positive transgenic *SpCIPK26*-OXP by reverse transcription-polymerase chain reaction (RT-PCR). Amplification of adenosine phosphoribosyl transferase *APT1* served as a loading control. L1, L2, L3, L4 and L5 represent five independent transgenic lines of *SpCIPK26*; WT, wild type *Arabidopsis*; M, marker.

Table S1. Primer sequence of real-time quantitative reverse transcription polymerase chain reaction (qRT-PCR).

Target	Forward Primer	Reverse Primer
RD29A	GATGACGAGCTAGAACCTGAAG	CCTTTGTCCCTGGTGGAATAA
RD29B	CCAGAACTATCTCGTCCCAAAG	GAAGCTAACTGCTCTGTGTAGG
ABF2	GCAACAGCAACAGCCAATC	CACAAGACCACCACCTCTTATC
CAT1	GGGAACAACTTCCCTGTATTCT	CCTCCAGTTCTCCTGAATGTG
UBQ10	CGGATCAGCAGAGGCTTATTT	GGGTGGATTCCTTCTGGATATTG
APT1	GCCAGTCGGGACAGTGAAATG	CACCAATAGCCAACGCAATAGG
SpCIPK26	AGCTCGCGCCTTCTTTACTAT	CCTTGCATAGATTGCTCAGGC
SpACT1	GATACGAGTAACGAACGGTGAT	CCCCCTCTCATTTTTATTACAC
